# Patterns of statin non-prescription in patients with established coronary artery disease: A report from a contemporary multicenter Japanese PCI registry

**DOI:** 10.1371/journal.pone.0182687

**Published:** 2017-08-17

**Authors:** Mitsuaki Sawano, Shun Kohsaka, Takayuki Abe, Taku Inohara, Yuichiro Maekawa, Ikuko Ueda, Koichiro Sueyoshi, Masahiro Suzuki, Shigetaka Noma, Yohei Numasawa, Hiroaki Miyata, Keiichi Fukuda, Kim G. Smolderen, John A. Spertus

**Affiliations:** 1 Department of Cardiology, Keio University School of Medicine, Tokyo, Japan; 2 Centre for Clinical Research, Keio University School of Medicine, Tokyo, Japan; 3 Department of Cardiology, Kawasaki-Municipal Kawasaki Hospital, Kanagawa, Japan; 4 Department of Cardiology, National Hospital Organization Saitama National Hospital, Saitama, Japan; 5 Department of Cardiology, Saiseikai Utsunomiya Hospital, Tochigi, Japan; 6 Department of Cardiology, Ashikaga Red Cross Hospital, Tochigi, Japan; 7 Department of Health Policy and Management, School of Medicine, Keio University, Tokyo, Japan; 8 Saint Luke's Mid America Heart Institute, University of Missouri-Kansas City School of Medicine, Kansas City, Missouri; University of Bologna, ITALY

## Abstract

Statin therapy is regarded as an effective medication to reduce cardiovascular events in patients at higher risk for future incidence of coronary artery disease. However, very few studies have been conducted to examine its implementation in non-Western real-world practice. In this study, we sought to describe statin prescription patterns in relation to patient characteristics in a Japanese multicenter percutaneous coronary intervention (PCI) registry as a foundation for quality improvement. We studied 15,024 patients that were prospectively enrolled in the Japan Cardiovascular Database-Keio interhospital Cardiovascular Study Registry from January 2009 to August 2014. The overall discharge statin non-prescription rate was 15.2%, without significant interhospital (MOR = 1.01) or annual differences (MOR = 1.13) observed. Hierarchical multivariable logistic regression analysis accounting for regional differences revealed that the presence of chronic kidney disease was associated with higher rates of statin non-prescription (OR 1.87, 95% confidence interval, 1.69–2.08, p value <0.001), and higher age (per 1-year increase) showed a trend for prescription of low-intensity statin (OR 1.00, 95% confidence interval, 1.00–1.01, p value = 0.045) within the subset of PCI patients (N = 4,853) enrolled after the year 2011. Our study indicates that patients with chronic kidney disease and elderlies may be the primary targets for maximizing the beneficial effect of statin therapy in post PCI patients.

## Introduction

Statin therapy is regarded as an essential medication to reduce cardiovascular events through its blood cholesterol lowering and anti-inflammatory effects. [1] [2–4] While minor differences exists for statin initiation thresholds and statin dosing, major society guidelines such as the American College of Cardiology/American Heart Association (ACC/AHA), [5] European Society of Cardiology/European Atherosclerosis Society (ESC/EAS),[6] National Lipid Association (NLA), [7] International Atherosclerosis Society (IAS),[8] and the U.S. Preventive Services Task Force (USPSTF),[9] describe a common understanding that statins are indeed effective upon risk reduction of cardiovascular events especially in those who possess high cardiovascular risk. In the secondary prevention setting, the 2013 ACCF/AHA guideline for the management of ST-elevation myocardial infarction gave a Class IB recommendation and the 2014 AHA/ACC guideline for the management of patients with non-ST-elevation acute coronary syndromes gave a Class IA recommendation for the use of moderate- or high-intensity statins. [10, 11] In fact, recent studies have provided evidence that higher intensity of statins do have a greater risk reducing effect compared to lower intensity statins. [12] [13] [14] Consequently, high or moderate intensity statin therapy at the timing of hospital discharge is considered an essential quality indicator for hospitals performing percutaneous coronary intervention (PCI) to established coronary artery disease patients. [15] [16] Despite these high recommendations, recent studies have suggested that statin underdosing is commonly observed in patients with high-risk profiles [17] [18]. These results indicate an important opportunity to modify current practice to reduce avoidable cardiovascular events although target patient or hospital characteristics for statin underdosing remain largely unknown in the real-world setting. Moreover, few studies have examined the implementation of statin therapy outside Western countries.[19, 20] [21]

In this study, we sought to describe prescription patterns of statin therapy after PCI and identify patient risk factors for statin non-prescription or low-intensity statin prescription in a contemporary Japanese percutaneous coronary intervention (PCI) registry as a foundation for quality improvement.

## Methods

### Study population

We studied 15,024 patients undergoing PCI in the Japan Cardiovascular Database-Keio interhospital Cardiovascular Studies (JCD-KiCS) registry from January 2009 to August 2014. The JCD-KiCS is a multicenter registry designed to collect clinical variables and outcome data from patients undergoing PCI. Dedicated clinical research coordinators assigned at each site enrolled the PCI patients consecutively. The clinical variables and in-hospital outcomes for the JCD-KiCS were defined in accordance with those specified for CathPCI Registry v4.1 (National Cardiovascular Data Registry (NCDR) in order to enable a direct comparison with the NCDR CathPCI Registry program. [22] Fifteen hospitals from Tokyo, Tochigi, Saitama, Chiba, and Kanagawa Prefecture in the Kanto area, Japan participated in this PCI registry. The participating hospitals were mostly large tertiary care referral centers (more than 200 beds; N = 12), but also included a few mid-sized satellite hospitals (less than 200 beds; N = 3). The average annual case-volume was 331 (ranging from 104 to 517) during the study period.

The participating hospitals were instructed to record and register data from consecutive hospital visits for PCI using an internet-based data collection system. Written informed consent was routinely obtained from patients before undergoing PCI. PCI with any commercially available coronary device was included. The quality of the data entered in the web-based data capture system were assured by an automatic validation system and reporting for data completeness, and through standardized education and training for the dedicated clinical research coordinators at each site (I.U.). Finally, the collected data were checked for completeness and internal consistency (S.K., Y.M.).

The Institutional Review Board of Keio University School of Medicine as well as each participating hospital approved the JCD-KiCS registry study protocol. The study was carried out in accordance with the approved guidelines. Before the launch of the registry, the objectives of the present study, its social significance, and an abstract were provided for clinical trial registration with the University hospital Medical Information Network (UMIN), which is recognized by the International Committee of Medical Journal Editors as an “acceptable registry” according to a statement issued in September 2004 (UMIN R000004736). UMIN is the largest and most versatile academic network information center for biomedical sciences in the world, and it is now considered as an indispensable information infrastructure for the Japanese medical community (http://www.umin.ac.jp/english/whatisumin.htm).

### Definitions of key variables

Previous history of CAD was defined as having a previous history of either myocardial infarction, PCI or CABG. Cardiogenic shock was defined as systolic blood pressure below 90 mmHg persisting over 30 minutes, the need for inotropes or mechanical circulatory support devices to maintain a systolic blood pressure of 90 mmHg or end-organ hypoperfusion state. Estimated glomerular filtration rate (eGFR) was calculated using the Modification of Diet in Renal Disease (MDRD) Study equation [23]. Presence of CKD was defined as an eGFR below 60 mL/min/1.73 m^2^. Additional data elements and definitions can be found at www.ncdr.com.

### Patient selection and exclusion criteria

**Fig 1:**
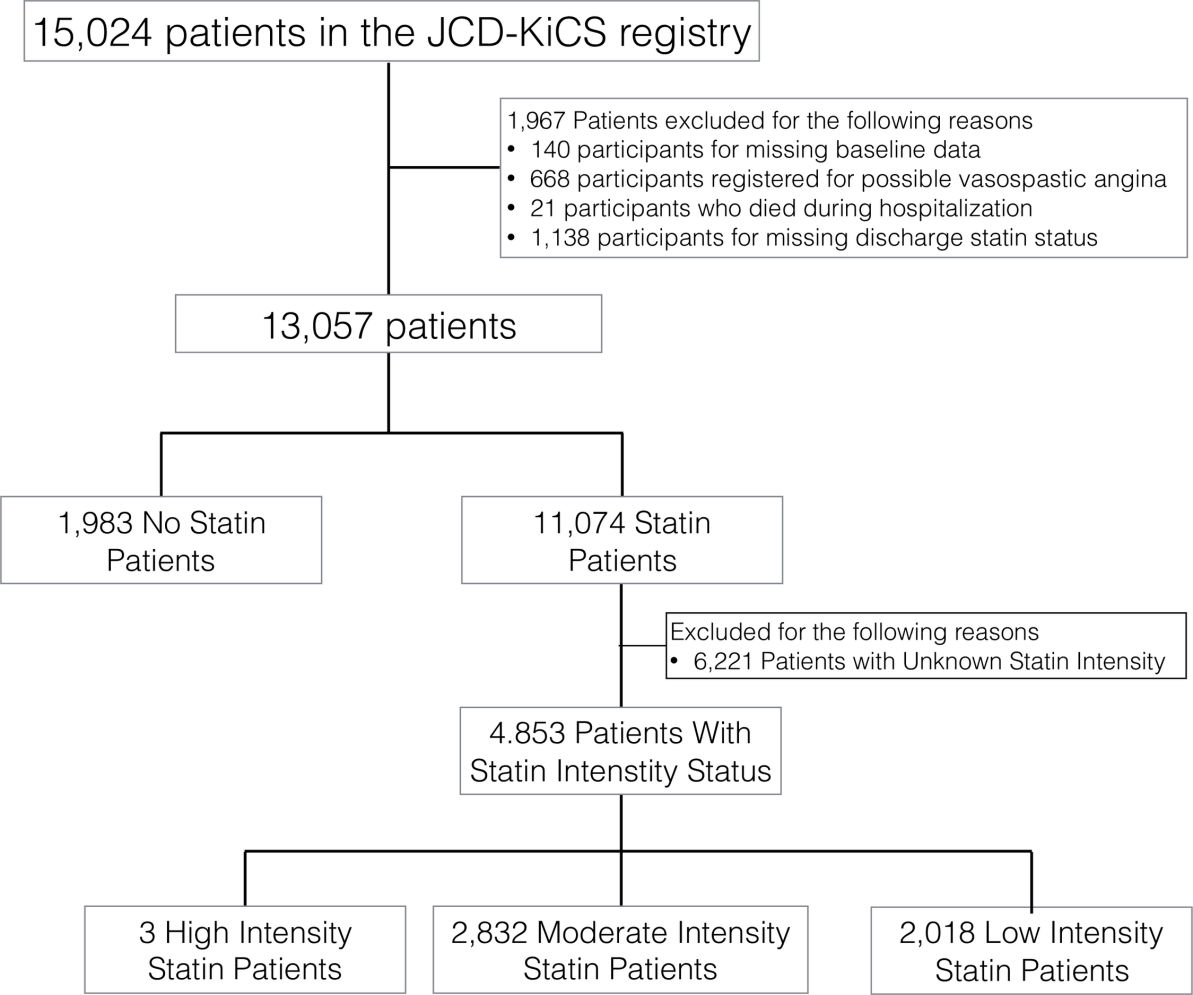
Study population for discharge statin prescription rate.

First, we compared the baseline demographics of patients discharged with and without statins (N = 13,057) (Fig 1). Patients with missing baseline data (N = 140), patients with possible vasospastic angina (N = 668), patients who died during the same hospitalization as the PCI procedure (N = 21), and patients with missing discharge statin status (N = 1,138) were excluded from the analysis (Fig 1 & S1 Table).

Second, within the subgroup of patients with complete statin intensity status (N = 4,853) (Fig 1) that began after the year 2011 as additional variables, statin intensity was classified according to the 2013 American College of Cardiology (ACC)/American Heart Association (AHA) guideline. High intensity statins were defined as atorvastatin 40-80mg/day and rosuvastatin 20mg/day, and moderate intensity statins were defined as atorvastatin 10-20mg/day, rosuvastatin 5-10mg/day, simvastatin 20-40mg/day, pravastatin 40mg/day, lovastatin 40mg/day, fluvastatin 40mg/day, pitavastatin 2-4mg/day. Any statins below these dosing were classified as low intensity statins [24].

### Statistical analysis

The Student’s t-test or the Wilcoxon rank-sum test was used to compare continuous variables of those with or without discharge statin at PCI discharge. The Chi-square test or Fisher’s exact test was used to compare categorical variables, as appropriate. To examine the extent of practice-level variation in statin use multivariable hierarchical logistic regression models were constructed to determine the median rate ratio (RR). These were two-level hierarchical models with the practice modeled as a random effect and patient covariates as fixed effects. [25] [26] The resulting median odds ratio (MOR) can be interpreted as the likelihood that two random practices would differ in treating theoretically identical patients with or without statins as well as treatment with low or moderate to high intensity statins after the PCI procedure. MOR is always above or equal to 1, with a MOR >1.20 suggesting significant practice-level variation. In addition, we examined whether patient-level predictors were stronger determinants of statin treatment than practice-level variation using a hierarchical multilevel logistic regression model. We included the following patient characteristic covariates to predict statin non-prescription: age, gender, and previous history of PCI and CKD due to their clinical significance from previous studies. Similarity, we included the following patient characteristic covariates to predict low-intensity statin prescription: age, gender, previous history of PCI and cerebrovascular disease due to their clinical significance from previous studies. A *p* value below 0.05 was considered to be statistically significant, unless mentioned otherwise in our analysis. All statistical analysis was performed using STATA version 13.1 (http://www.stata.com).

## Results

**Fig 2:**
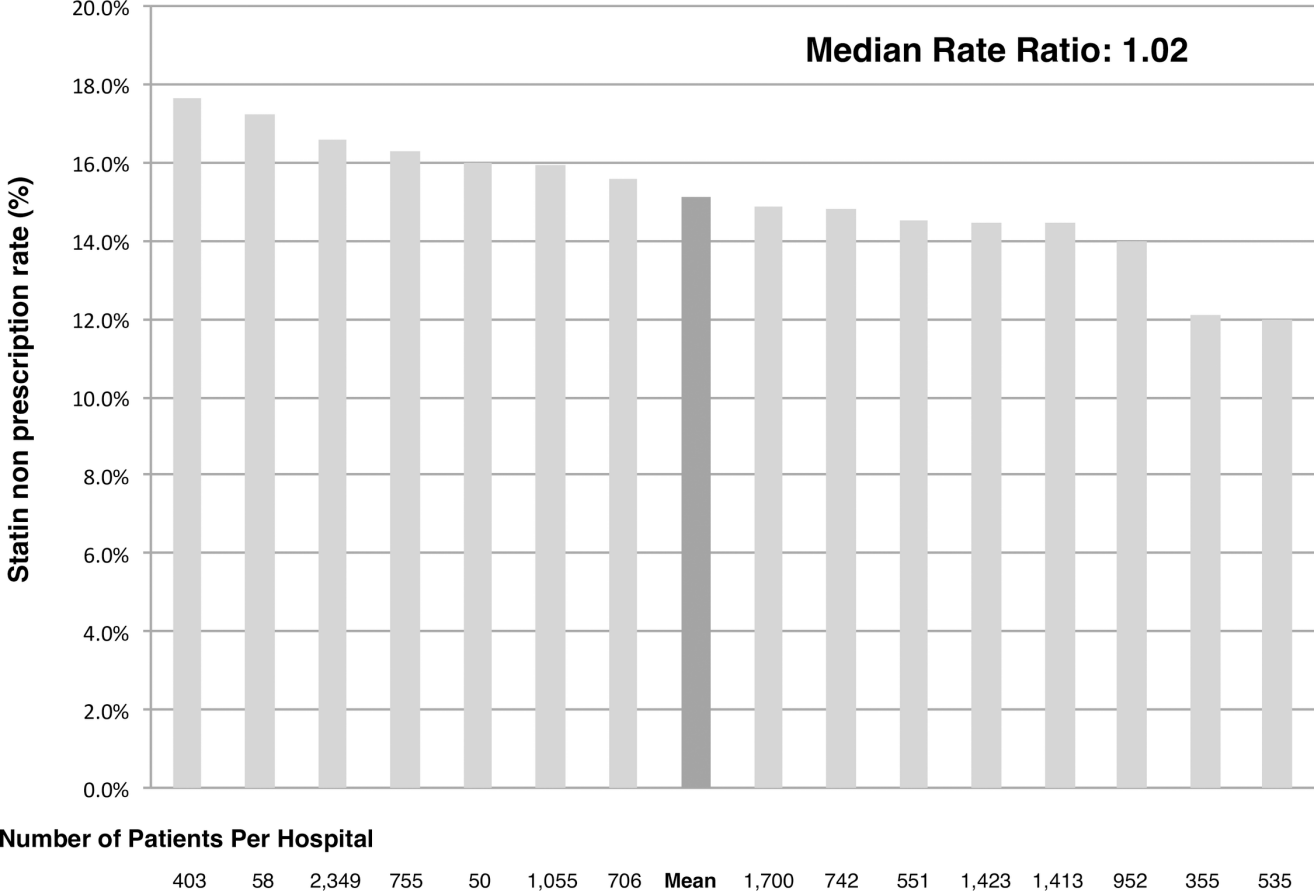
Statin non-prescription rate among participating hospitals.

The current study population was consisted of 13,057 PCI patients with a mean age of 68.0 ± 10.9 years, 20.6% female, an average BMI of 24.2 ±3.6, 37.3% with a history of PCI, 5.5% with a history of CABG and 46.9% presented to the PCI hospital with acute coronary syndrome (Table 1). Overall, 15.2% (N = 1,983) of the patients did not receive statin therapy at the timing of discharge. Patients without discharge statins had similar baseline characteristics and CAD presentation as well as serum cholesterol levels upon admission compared with those who were on statins, although presence of CKD was frequently observed in the statin non-prescription group (39.9% vs 26.6%, *P* value <0.001)(Tables 1 & 2). Discharge prescription rate for other key medications such as aspirin (98.7% vs 96.1%, *P* value <0.001), clopidogrel (91.2% vs 86.1%, *P* value <0.001), renin angiotensin aldosterone system inhibitors (66.3% vs 57.1%, *P* value <0.001) and beta-blockers (70.1% vs 51.0%, *P* value <0.001) were significantly lower in the statin non-prescription group while a greater proportion of patients were on warfarin in the same group (9.6% vs 12.1%, *P* value <0.001). Statin non-prescription rate ranged from a minimum of 11.9% to a maximum of 18.0% among participating hospitals (MOR = 1.01) (Fig 2) and a minimum of 14.3% to a maximum of 15.6% among patient registration year (MOR = 1.13).

**Table 1:** Baseline characteristics of patients with and without discharge statins.

		Overall		No Statin		Statin		
		N = 13,024		N = 1,983		N = 11,078		P value
Age (mean, SD)		68 ±10.9		68 ±10.6		68.1±10.9		0.91
Female		2557	20.6%	410	21.7%	2147	20.4%	0.23
Body mass index (mean,SD)		24.2 ±3.6		24.1 ±13.5		24.2±3.7		0.42
Medical History								
	Myocardial infarction	3062	24.6%	452	23.9%	2610	24.9%	0.34
	Heart failure	1101	8.9%	170	9.0%	931	8.9%	0.9
	PCI	4650	37.3%	695	36.7%	3955	37.6%	0.45
	CABG	672	5.5%	96	5.1%	576	5.5%	0.47
Diabetes mellitus		5137	41.9%	800	42.4%	4337	41.5%	0.64
Chronic Kidney Disease		3701	28.3%	781	39.9%	2920	26.6%	<0.001
	Hemodialysis	578	4.4%	76	4.0%	502	4.8%	0.14
Cerebrovascular disease		1108	8.5%	177	9.4%	931	8.9%	0.51
Peripheral vascular disease		1040	8.0%	163	8.6%	877	8.4%	0.75
Chronic lung disease		381	2.9%	52	2.8%	329	3.1%	0.36
Hypertension		9268	70.9%	1405	74.4%	7863	75.2%	0.44
Smoking		4181	32.0%	624	33.1%	3557	34.1%	0.37
Dyslipidemia		8173	62.5%	1239	65.7%	6934	66.4%	0.56
Family history of CAD		1440	11.0%	223	11.9%	1217	11.8%	0.86
Cancer		487	3.7%	68	3.8%	419	4.1%	0.45
Cardiogenic shock		552	4.2%	86	4.3%	466	4.2%	0.81
Acute coronary syndrome		6022	46.1%	923	46.5%	5099	46.1%	0.3
Discharge medications								
	Aspirin	12894	98.7%	1912	96.1%	10982	99.1%	<0.001
	Clopidogrel	11924	91.2%	1712	86.1%	10212	92.2%	<0.001
	RAAS inhibitor	8659	66.3%	1136	57.1%	7523	67.9%	<0.001
	Beta-blockers	9166	70.1%	1014	51.0%	8152	73.6%	<0.001
	Warfarin	1255	9.6%	241	12.1%	1014	9.2%	<0.001

Abbreviations: CAD; Coronary artery disease, SD; Standard deviation, PCI; Percutaneous coronary intervention, CABG; Coronary arterial bypass graft, LDL; Low-density lipoprotein, HDL; High-density lipoprotein, RAAS; Renin angiotensin aldosterone system

**Table 2:** Lipid profiles of patients with and without discharge statins.

	Overall	No Statin	Statin	
	N = 13,068	N = 1,990	N = 11,078	P value
Total cholesterol	182±43	178±39	182±43	0.08
LDL	105±37	105±30	105±37	0.95
HDL	47±14	47±14	47±14	0.72
Triglyceride	125 (86–183)	123(86–172)	127(88–186)	0.07

Total cholesterol, LDL, HDL are presented as mean ± standard deviation

Triglyceride is presented as median and first and third quartiles.

Abbreviations: LDL; Low-density lipoprotein, HDL; High-density lipoprotein

**Fig 3:**
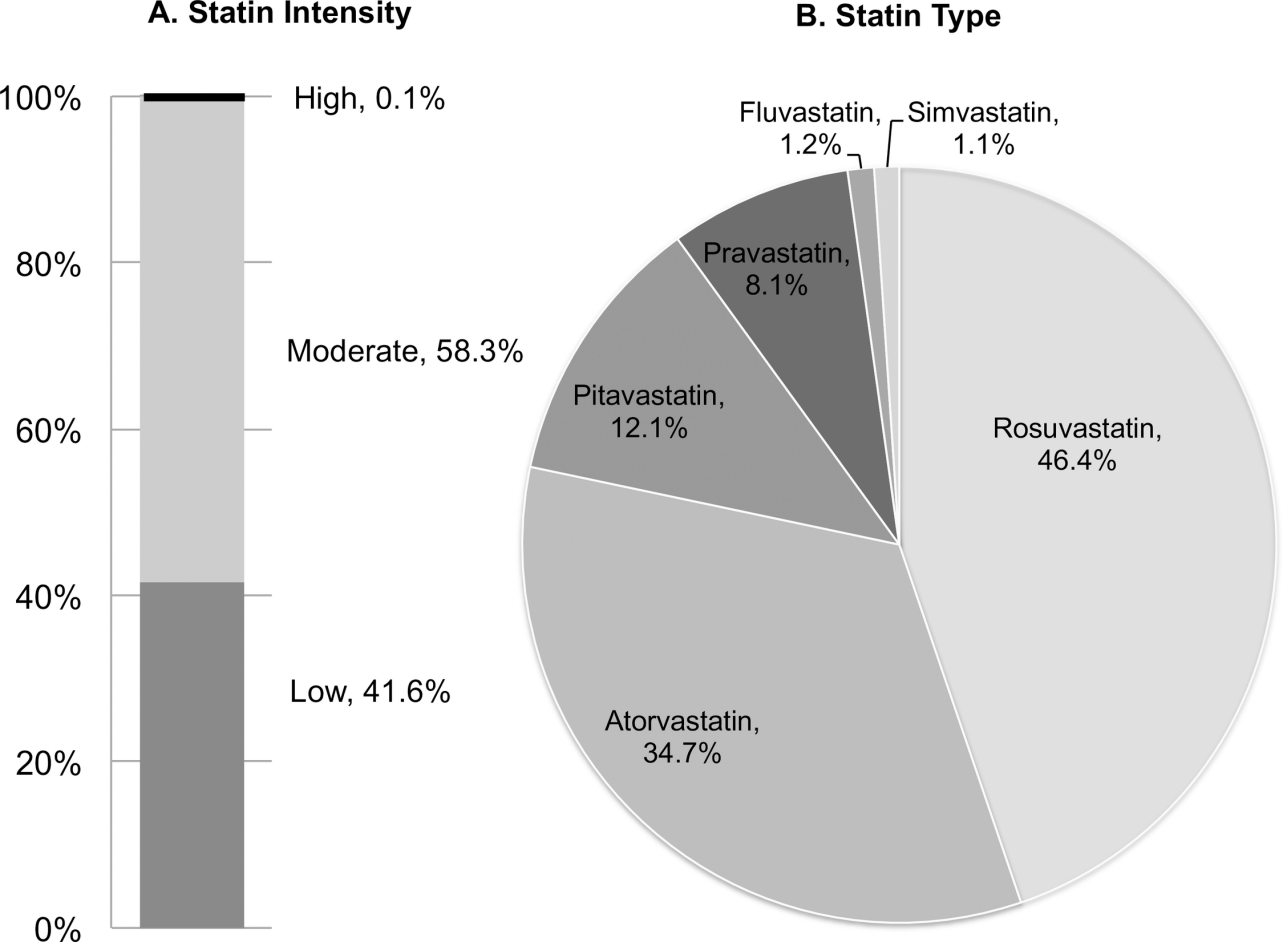
Discharge statin intensity and statin type.

**Fig 4:**
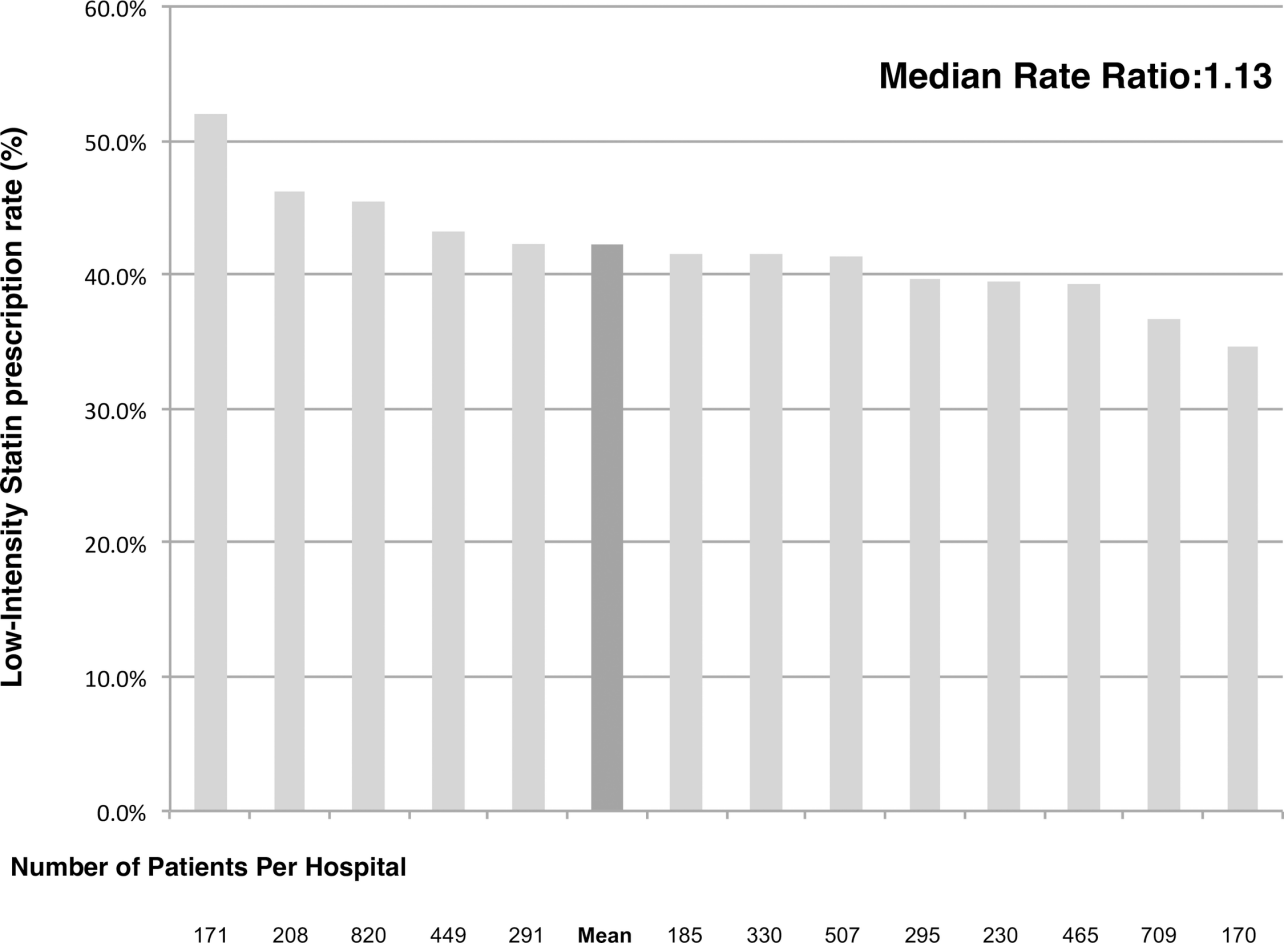
Low-intensity statin prescription rate among participating hospitals.

The second part of our analysis was performed within the subgroup of patients with full description of statin type and dosing (N = 4,845); 41.5% (N = 2,018) of the patients were on low-intensity statins and 58.4% were on moderate to high-intensity statins at the timing of discharge (Fig 1 & Table 3). Merely three patients received high-intensity statins within this Japanese study population. Rosuvastatin (46.4%) and atorvastatin (34.7%) were the two most favorably prescribed brands of statins in this population (Fig 3). Among the participating hospitals with more than a hundred cases registered within this subgroup population, the low-intensity statin prescription rate ranged from a minimum of 34.7% to a maximum of 52.0% (MOR = 1.13) (Fig 4) and a minimum of 39.8% to a maximum of 44.2% among patient registration year after the year 2011 (MOR = 1.12).

**Table 3:** Baseline characteristics of patients with low-intensity and moderate or high-intensity discharge statins.

		Overall		Low- Intensity Statin		Moderate-High Intensity Statin		
		N = 4,845		N = 2,016		N = 2,837		P value
Age (mean, SD), y		68.1±10.9		68.4±10.9		67.8±11.0		0.05
Female		943	19.40%	403	20.00%	540	19.10%	0.38
Body mass index (mean, SD)		24.2±3.7		24.2±3.7		24.3±3.6		0.14
Medical History								
	Myocardial infarction	1126	23.20%	475	23.60%	651	23.00%	0.57
	Heart failure	391	8.10%	173	8.60%	218	7.70%	0.25
	PCI	1721	35.50%	743	36.90%	978	34.60%	0.07
	CABG	244	5.00%	112	5.60%	132	4.70%	0.15
Diabetes mellitus		1909	39.30%	812	40.30%	1097	38.80%	0.24
Chronic Kidney Disease		1342	27.70%	561	27.80%	781	27.60%	0.87
	Hemodialysis	205	4.20%	90	4.50%	115	4.10%	0.47
Cerebrovascular disease		403	8.30%	186	9.20%	217	7.70%	0.046
Peripheral vascular disease		378	7.80%	167	8.30%	211	7.50%	0.26
Chronic lung disease		145	3.00%	63	3.10%	82	2.90%	0.63
Hypertension		3478	71.70%	1426	70.70%	2052	72.50%	0.27
Smoking		1532	31.60%	625	31.00%	907	32.10%	0.53
Dyslipidemia		3095	63.80%	1263	62.60%	1832	64.80%	0.19
Family history of CAD		512	10.60%	207	10.30%	305	10.80%	0.63
Cardiogenic shock		206	4.20%	85	4.20%	121	4.30%	0.93
Acute coronary syndrome		2272	46.80%	942	46.70%	1330	47.00%	0.83

Abbreviations: CAD; Coronary artery disease, SD; Standard deviation, PCI; Percutaneous coronary intervention, CABG; Coronary arterial bypass graft

Finally, hierarchical multivariable logistic regression analysis accounting for practice variability revealed that the presence of chronic kidney disease was associated with higher rates of statin non-prescription (OR 1.87, 95% confidence interval, 1.69–2.08, p value <0.001) after adjusting for age, previous history of PCI (S2 Table), whereas higher age (per 1-year increase) was associated with prescription of low-intensity statin (OR 1.00, 95% confidence interval, 1.00–1.01, p value = 0.045) within the previously mentioned subgroup study population after adjusting for gender, previous history of PCI and presence of CKD (S3 Table).

## Discussion

Our current study showed that more than 15% of PCI patients were discharged without statin therapy despite a strong recommendation from various practice guidelines. Moreover, low-intensity statins was frequently observed in those who did receive statins at the timing of discharge. The proportion of statin non-prescription and low-intensity statin prescription were similar across the participating sites and throughout patient enrollment year. These results suggest that patient characteristics such as the presence of CKD and higher age can be potential targets to enhance the quality of care for PCI patients in Japan through maximizing statin therapy.

In our dataset, the overall discharge statin non-prescription rate was comparable or higher than that reported in previous Western/non-Western databases. Recently, Pokharel et al reported the current trend of moderate-intensity to high-intensity statin therapy usage in the United States, before and after the publication of the 2013 ACC/AHA guideline from the American College of Cardiology National Cardiovascular Data Registry’s Practice Innovation and Clinical Excellence (PINNACLE) Registry. [27] The authors reported a 67.0% moderate-intensity to high-intensity statin prescription rate in ASCVD patients despite a strong recommendation in the new guideline. Similarly, Rodriguez et al also described the current statin prescription status of ASCVD patients treated in the Veterans Affairs health care system from April 1, 2013, to April 1, 2014 and found that 18.2% did not receive any statins resulting in a higher mortality rate compared to other patients on low to high intensity statins. [28] In the European nations underutilization of discharge statin therapy has been identified in the EUROASPIRE IV survey that reported a 9.6% of CHD patients who were not on statins at discharge. [29] In Japan, Natsuaki et al reported from the CREDO-Kyoto registry cohort-2 that the discharge statin prescription rate was low as 49.0% in patients undergoing coronary revascularization although this data was derived during 2005 to 2007 and may best represent the current practice in provided in Japan. [30] Similarly, Kaneko et al reported from a single-center study that nearly 40% of stable CAD patients did not receive statin therapy upon discharge and was indeed associated with a higher risk of three-year major cardiovascular events. [31] Our current data was derived from 2009 to 2014 that is likely to represent current practice patterns and were also collected from various hospitals situated in the neighboring areas of Tokyo.

Notably, an exceptionally high statin non-prescription rate was observed in CKD patients of our registry. In fact, the odds of receiving a statin at the timing of discharge were halved when CKD was present in the current Japanese study. Prescribing a fixed dose of statins in CKD patient has been controversial since these patients are perceived to be the most vulnerable to experience statin adverse effects especially when receiving higher intensity statins. The 2013 ACC/AHA Guideline on the Treatment of Blood Cholesterol to Reduce Atherosclerotic Cardiovascular Risk in Adults [15] support the prescription of high-intensity statins therapy in adults with clinical CVD and CKD if not on hemodialysis. This recommendation is supported by a prospective observational study and meta-analysis showing the beneficial effect of statin therapy to reduce fatal cardiovascular events as well as non-fatal cardiovascular events. [32] [33] [34] On the contrary, a recent study conducted by Acharya et al suggest that statin use is associated with aggravating effect on renal function, thus it is difficult to conclude whether or not statin use is effective universally in this population. [35] The local Japan Atherosclerosis Society Guidelines for Prevention of Atherosclerotic Cardiovascular Diseases 2012 also gives similar recommendations for using statins in this particular population. [36] As for patients who are already on hemodialysis, the 2013 ACC/AHA guideline does not give any statements and the decision is largely left to the physician’s discretion. [24] Interestingly, in our dataset, hemodialysis patients had a higher statin prescription rate compared to CKD patients not on hemodialysis although they represent only four percent of the entire cohort. This may be due to the physician’s belief that hemodialysis patients would tolerate the medication better by receiving mechanical excretion of drug and metabolites without affecting renal function. The outcome of this practice pattern is yet to be determined when further long-term outcome data is available.

Suboptimal dosing of discharge statins is also a problem along with statin non-prescription. In our study, only 3 (0.1%) patients were prescribed with high-intensity statins while 41.6% were on low-intensity statins that may lead to cardiovascular events if not titrated up to higher dosing. Importantly, Rodriguez et al. described that high-intensity statins are indeed associated with a small but significant survival advantage compared with moderate-intensity statins, even among older adults. [14] In the current 2013 ACC/AHA guideline, high-intensity statin therapy is recommended in patients with established CAD under the age of 75 and moderate intensity statins above the age of 75 if tolerated. [24] A study of ACS patients from the Get With The Guidelines database showed that 38.3% of the population was treated with high intensity statins as compared with 0.1% in the current Japanese population. [37] However it is worth mentioning that moderate intensity statins were prescribed in 58.3% of the study population with 26.8% of the population aging above 75. There are several factors that could have influenced cholesterol treatment patterns for post PCI patients in Japan; 1) the solely approved high-intensity statin in Japan is rosuvastatin given at 20mg per day, 2) higher sensitivity towards statins in East Asians leading to the anticipation of increased risk for statin intolerance and 3) relatively large proportion of elderly PCI patients with more comorbidities. East Asians are recognized to hold a higher risk for statin intolerance especially when additional factors such as older age, female gender, low BMI and CKD is present. [38, 39] Moreover, since a larger proportion of Japanese PCI patients are in their eighties or nineties compared to the Western PCI population, more patients are likely to hold these risks and the number of elderly CAD population is expected to grow over the coming two to three decades in this region of the world [22, 40]. Since there is no clear-cut answer for statin dosing in elderly patients, more evidence through randomized clinical trials and high quality observational research with a patient centered, tailored approach is warranted in this unique population. [41]

Recently, two new classes of non-statin blood-lowering medications have been shown to improve clinical outcomes in addition to maximally tolerated dose of statins; ezetimibe and proprotein convertase subtilisin/kexin type 9 (PCSK9) inhibitors.[42–44] These medications may play a key role in treating patients with familial hypercholesterolemia and those with statin intolerance despite holding a high CVD risk but the cost associated with these medications are also a risk that the patient and physician need to take into consideration. Our study indicates that there is room for improvement to enhance the quality of post PCI care by utilizing contemporary and cost efficacious resources such as statin therapy. To meet this goal, elderlies and CKD patients who underwent PCI may be the ideal target to improve care in this real-world registry.

## Limitations

There are several limitations to our current study. First, our study had the inherent limitations of any nonrandomized observational research. These results are derived from a multicenter cohort registry that cannot indefinitely clarify the cause and effect relationship between the exposure of interest and outcomes even after rigorous adjustment for possible confounders. Second, key factors that may have affected statin non-prescription such as medication status prior to PCI admission, previous intolerance to statins, statin allergy, patient preferences, socioeconomic and education level were not collected in this registry although the impact of socioeconomic status may be smaller compared to other countries with much disparity since Japan is known to have a relatively large proportion of middle-class income households reinforced with a universal health care system. Third, the current registry did not collect details of post-discharge follow-up clinic, especially data on post-discharge statin initiation or titrations, however, we believe that the number of these patients are small and do not affect the overall results shown in our current analysis. Fourth, long-term outcome data on cardiovascular death or unplanned hospitalization for revascularization were not available in this current database. Further follow-up data is necessary to evaluate the relationship of discharge statin non-prescription, dose-intensity and subsequent clinical hard endpoints. Fifth, although evaluated within a large prospective multicenter registry, our current data represents the real-world prescription of statins in Japanese PCI patients and may not be generalizable in Western countries where the incidence and demographics of coronary artery disease patients differ largely. However, given the fact that we are still at the dawn of implementing high-intensity statins after PCI when looking at a global scale, our results may be of value in countries struggling to improve quality of care by targeting patients with high-risk characteristics based on current/previous cardiology practice.

## Conclusions

The findings of this study suggest that discharge statin non-prescription rates following PCI in a Japanese context are considerably high, especially in those with chronic kidney disease. In addition, in contrast to the recommendation of using high-intensity statins in established CAD patients, low-intensity statins prescription was frequently observed in a subgroup of post PCI patients with complete statin intensity status. Long-term data on the subsequent incidence of recurrent coronary events as a function of these observed treatment practices is warranted.

## Supporting information

S1 Table Baseline demographics of the study population with missing statin status. (DOCX)

S2 Table Hierarchal logistic regression analysis predicting discharge statin non-prescription accounting for hospital differences. (DOCX)

S3 Table Hierarchal logistic regression analysis predicting discharge low-intensity statin prescription accounting for hospital differences. (DOCX)
