# Supplementary material for: Patterns of statin non-prescription in patients with established coronary artery disease: A report from a contemporary multicenter Japanese PCI registry
Source: PLoS One. 2017 Aug 17;12(8):e0182687. doi: 10.1371/journal.pone.0182687 (PMC5560610; doi:10.1371/journal.pone.0182687)
Supplement: S1 Table — (DOCX) [file pone.0182687.s001.docx]

S1 Table. Baseline demographics of the study population with missing statin status

|  |  | Overall | | Missing Statin Status | |  |
| --- | --- | --- | --- | --- | --- | --- |
|  |  | N=13,057 | | N= 1,138 | | P value |
| Age | | 68 ±10.9 | | 68.2±10.8 | | 0.29 |
| Female | | 2690 | 20.6% | 173 | 15.2% | 0.25 |
| Body mass index | | 24.2 ±3.6 | | 23.8 ±3.3 | | 0.47 |
| Medical History | |  |  |  |  |  |
|  | Myocardial infarction | 3212 | 24.6% | 716 | 62.9% | <0.001 |
|  | Heart failure | 1162 | 8.9% | 402 | 35.3% | <0.001 |
|  | PCI | 4870 | 37.3% | 954 | 84% | <0.001 |
|  | CABG | 718 | 5.5% | 52 | 4.6% | 0.70 |
| Diabetes mellitus | | 5471 | 41.9% | 534 | 46.9% | 0.55 |
| Chronic Kidney Disease | | 3695 | 28.3% | 279 | 24.5% | <0.001 |
|  | Hemodialysis | 575 | 4.4% | 69 | 6.1% | 0.61 |
| Cerebrovascular disease | | 1110 | 8.5% | 147 | 12.9% | 0.43 |
| Peripheral vascular disease | | 1045 | 8.0% | 34 | 3.0% | 0.27 |
| Chronic lung disease | | 379 | 2.9% | 34 | 3.0% | 0.99 |
| Hypertension | | 9257 | 70.9% | 819 | 72.0% | 0.95 |
| Smoking | | 4178 | 32.0% | 676 | 59.4% | <0.001 |
| Dyslipidemia | | 8161 | 62.5% | 751 | 66.0% | 0.97 |
| Family history of CAD | | 1436 | 11.0% | 104 | 9.1% | 0.58 |
| Cancer | | 483 | 3.7% | 0 | 0.0% | 0.28 |
| Cardiogenic shock | | 548 | 4.2% | 42 | 3.7% | 0.35 |
| Acute coronary syndrome | | 6019 | 46.1% | 157 | 13.8% | <0.001 |

Abbreviations: PCI=Percutaneous coronary intervention; CABG= Coronary artery bypass graft; CAD= coronary artery disease
