# Supplementary material for: Patterns of statin non-prescription in patients with established coronary artery disease: A report from a contemporary multicenter Japanese PCI registry
Source: PLoS One. 2017 Aug 17;12(8):e0182687. doi: 10.1371/journal.pone.0182687 (PMC5560610; doi:10.1371/journal.pone.0182687)
Supplement: S2 Table — (DOCX) [file pone.0182687.s002.docx]

S2 Table. Hierarchal logistic regression analysis predicting discharge statin non-prescription accounting for hospital differences

|  | Covariates within model | Odds ratio (95%CI) | p value |
| --- | --- | --- | --- |
| Model 1 | |  |  |
|  | Age　(per 1-year increase) | 1.00 (1.00-1.01) | 0.21 |
|  | CKD | 0.54 (0.49-0.60) | <0.001 |
| Model 2 | |  |  |
|  | Age　(per 1-year increase) | 1.00 (1.00-1.01) | 0.2 |
|  | Gender | 0.94 (0.83-1.07) | 0.33 |
|  | CKD | 0.53 (0.47-0.59) | <0.001 |
| Model 3 | |  |  |
|  | Age　(per 1-year increase) | 1.00 (1.00-1.01) | 0.19 |
|  | Gender | 0.93 (0.82-1.060 | 0.29 |
|  | Previous PCI | 1.03 (0.93-1.15) | 0.52 |
|  | CKD | 0.53 (0.47-0.59) | <0.001 |

Abbreviations: PCI=Percutaneous coronary intervention; CKD=Chronic Kidney Disease
