# Supplementary material for: Patterns of statin non-prescription in patients with established coronary artery disease: A report from a contemporary multicenter Japanese PCI registry
Source: PLoS One. 2017 Aug 17;12(8):e0182687. doi: 10.1371/journal.pone.0182687 (PMC5560610; doi:10.1371/journal.pone.0182687)
Supplement: S3 Table — (DOCX) [file pone.0182687.s003.docx]

S3 Table. Hierarchal logistic regression analysis predicting discharge low-intensity statin prescription accounting for hospital differences

|  | Covariates within model | Odds ratio (95%CI) | p value |
| --- | --- | --- | --- |
| Model 1 | |  |  |
|  | Age　(per 1-year increase) | 1.00 (1.00-1.01) | 0.053 |
| Model 2 | |  |  |
|  | Age　(per 1-year increase) | 1.01 (1.00-1.01) | 0.04 |
|  | Previous PCI | 1.12 (0.99-1.27) | 0.06 |
| Model 3 | |  |  |
|  | Age　(per 1-year increase) | 1.01 (1.00-1.01) | 0.045 |
|  | Previous PCI | 1.13 (1.00-1.27) | 0.05 |
|  | Cerebrovascular disease | 1.21 (0.99-1.49) | 0.07 |

Abbreviations: PCI=Percutaneous coronary intervention
